# Case Report: Papillary thyroid carcinoma in Goltz–Gorlin syndrome

**DOI:** 10.3389/fendo.2023.1243540

**Published:** 2023-10-04

**Authors:** Flavia Costanza, Giampaolo Papi, Stefania Corrado, Alfredo Pontecorvi

**Affiliations:** ^1^ Endocrinology, Diabetology and Internal Medicine Unit, Catholic University of the Sacred Heart, Rome, Italy; ^2^ Endocrinology Unit, Azienda USL Modena, Modena, Italy; ^3^ Department of Pathology and Laboratory Medicine, University of Modena and Reggio Emilia, Modena, Italy

**Keywords:** papillary thyroid carcinoma, Goltz-Gorlin syndrome, focal dermal hypoplasia, X-linked disorders, rare genetic syndromes

## Abstract

Goltz–Gorlin syndrome (GGS), also known as focal dermal hypoplasia, is a rare X-linked disorder caused by pathogenic variants in the PORCN gene and characterized by several abnormalities, including skin and limb defects, papillomas in multiple organs, ocular malformations, and mild facial dysmorphism. To date, only approximately 300 cases have been described in the literature. A 16-year-old female patient, born with multiple congenital dysmorphisms consistent with GGS and confirmed by genetic exam, was referred to our outpatient clinic for the workup of a thyroid nodule. A thyroid ultrasound showed a bilateral nodular disease with a 17-mm large hypoechoic nodule in the right lobe. Cytological exam of fine needle aspiration biopsy was suspicious for malignancy. Thus, she underwent total thyroidectomy plus lymphadenectomy of the right central compartment. A histological exam disclosed a papillary thyroid carcinoma (PTC) with lymph node micrometastases. Radioiodine (131-Iodine) therapy was performed. At 3- and 6-month follow-up, the patient did not present either ultrasound or laboratory PTC recurrence. To our knowledge, we report the first case of PTC in a patient with GGS. Since thyroid cancer is rare among children and adolescents, we hypothesize that the PORCN pathogenic variant could be responsible for tumor susceptibility. We also provide an overview of the clinical findings on GGS patients already reported and discuss the possible pathogenetic mechanism that may underlie this rare condition, including the role of PORCN in tumor susceptibility.

## Introduction

Goltz–Gorlin syndrome (GGS), also known as focal dermal hypoplasia (FDH), is an X-linked dominant, multiple congenital dysmorphic disorder caused by pathogenic variants in the PORCN gene. It is characterized by numerous abnormalities, which show a segmental arrangement reflecting functional X-chromosome mosaicism (1). GGS is a very rare disorder; until now, only approximately 300 cases have been described in the literature. Clinical manifestations are extremely variable among individuals and involve different organs and tissues (1) (**Table 1**). Patchy skin atrophy with fat herniation following the lines of Blaschko is a typical finding in this syndrome and it is reported at birth in nearly 75% of cases. Other dermal features involve patchy alopecia, atrophic areas, susceptibility to recurrent erosions, hyperpigmented freckling, and telangiectasias within hypoplastic lesions. Widely distributed papillomas are reported in nearly two-thirds of affected individuals. Nail changes are common findings, including longitudinal ridging, micronychia, and anonychia. Children with GGS may show distinctive facial features already at birth, such as a pointed chin, small ears, notched nostrils, and a slight difference in the size and shape of the right and left sides of the face. Oral tissue defects occur in most of the subjects. They can manifest hard vertical dental enamel grooving, peg-shaped teeth, enamel hypoplasia, and cleft lip or palate. Moreover, intraoral lipomas or papillomas are common. Papillomas can also develop in the nose, pharynx, larynx, trachea, esophagus, and stomach. In almost half of cases, limb abnormalities are present, including leg length discrepancy, congenital absence of foot and ankle, giant cell tumor of the bone, osteopathia striata, syndactyly, and ectrodactyly. Children affected by GGS usually have difficulty with feeding and swallowing and have chewing problems, gastroesophageal reflux, gastroparesis, and constipation. Abdominal defects such as diaphragmatic hernia, hiatal hernia, umbilical hernia, and omphalocele have been reported. Three-fourths of patients manifest ophthalmologic problems, such as coloboma, microphthalmia, cataracts, nystagmus, and strabismus, as well as papillomas of the eyelid and conjunctiva. Central nervous system pathological findings can include hydrocephalus, agenesis of the corpus callosum, sensorineural hearing loss, and Arnold-Chiari malformation. Behavioral and emotional problems can show during the school term. Urogenital anomalies are commonly observed: in affected girls, labia minora hypoplasia, short perineum body, bicornuate uterus, unilateral absent kidney, hypoplastic kidney, fused kidney, and cystic renal dysplasia have been reported. Asymmetry of the nipples may occur in one-third of individuals.

**Table 1:** Clinical features of Goltz–Gorlin Syndrome: overview of the literature.

Head and neck	Respiratory	Chest	Abdominal	Genito-urinary	Skeletal	Skin, nails, hair	Neurological, eyes and, ears
**Head** Microcephaly **Face** Facial asymmetry Pointed chin **Nose** Narrow nasal bridge Broad nasal tip Notched nasal alae **Mouth** Lips papillomas Cleft lip Cleft palate **Teeth** Hypodontia Oligodontia Enamel hypoplasia Delayed eruption Malocclusion Notched incisors Odontogenic keratocysts Gingivas papillomas	**Nose** Papillomatosis **Larynx** Papillomatosis **Pharynx** Papillomatosis **Trachea** Papillomatosis	**Ribs, Clavicles** Midclavicular aplasia Midclavicular hypoplasia Rib hypoplasia **Breasts** Asymmetric breast Supernumerary nipples Nipple hypoplasia Asymmetric nipples **Diaphragm** Diaphragmatic hernia	**External Features** Umbilical hernia Omphalocele Diastasis recti **Gastrointestinal** Hiatus hernia Gastroesophageal reflux Gastroparesis Constipation Anteriorly displaced anus Intestinal malrotation Esophageal papillomas Stomach papillomas	**External Genitalia (Male)** Inguinal hernia **External Genitalia (Female)** Clitoral hypoplasia Labial hypoplasia **Internal Genitalia (Male)** Cryptorchidism **Internal Genitalia (Female)** Bicornuate uterus **Kidneys** Horseshoe or fused kidney Hydronephrosis Absent kidney Hypoplastic kidney Cystic renal dysplasia **Ureters** Bifid ureter **Perineum** Short perineum body	**Skull** Asymmetric skull **Spine** Scoliosis Spina bifida occulta **Pelvis** Failure of pubic bone fusion Congenital hip dislocation **Limbs** Osteopathia striata **Hands** Short phalanges Short metacarpal **Feet** Short metatarsal Hypoplastic digits Missing toes **Fingers** Ectrodactyly Syndactyly Brachydactyly Polydactyly	**Skin** Linear or reticular hyperpigmentation Skin atrophy Telangiectasia Localized cutaneous deposits of superficial fat Arborescent papillomas Hypoplastic fingertip epidermal ridges Hidrocystomas **Nails** Dystrophic nails (spooned, grooves) Micronychia Absent fingernails Absent toenails **Hair** Sparse hair Brittle hair Patchy alopecia	**Central Nervous System** Mental retardation Myelomeningocele Hydrocephalus Agenesis of corpus callosum Arnold-Chiari malformation **Eyes** Iris coloboma Choroidoretinal coloboma Aniridia Microphthalmia Anophthalmia Ectopia lentis Optic atrophy Nystagmus Strabismus Decreased visual acuity Eyelid papillomas **Ears** Low-set ears Narrow auditory canals Hearing loss, mixed

The prognosis of patients affected by GGS depends on the degree of systems impairment, so close development monitoring and multidisciplinary team management improve the outcomes.

Despite several reviews of the literature over the years, alterations of the endocrine system related to this syndrome have rarely been reported. Only one study (2) suggests possible growth hormone deficiency in a minority of patients. With regard to the thyroid gland, just one case of a patient affected by GGS with a thyroid nodule was described, by Disdier in 1998. To our knowledge, we report the first case of papillary thyroid carcinoma (PTC) in a patient affected by GGS.

## Case description

A 16-year-old female patient was referred to our outpatient clinic for the management of a thyroid nodule in June 2022. She was born full-term in 2006, with multiple congenital dysmorphisms, including hands and feet syndactyly and oligodactyly, right eye coloboma, right microphthalmia, right optic nerve hypoplasia, ridged malformed nails, skin telangiectasias, and mild facial dysmorphism. For these clinical findings, the pediatrician suspected she could be affected by GGS. Subsequently, in 2007, a DNA sample was taken and sent to an international genetic center for the PORCN mutation analysis. As the PORCN gene encodes in 15 exons of the longest splice variant, isoform D, a 1,386-bp cDNA that is translated into a protein of 461 amino acids, the coding exons, including the intron–exon boundaries, were amplified from genomic DNA. The PCR products were sequenced on both strands of the DNA. The PCR products of exon 14 were subjected to single-strand conformation analysis. The presence of a point mutation encountered was searched in a sample of 100 unrelated individuals to exclude that it represented a polymorphism. The DNA sequence analysis of the PORCN gene from our patient detected in exon 14, in addition to a wild-type sequence, a *de novo* heterozygous missense mutation c.1250T>C; p.F417S. A mutation was confirmed by single-strand conformation analysis. Furthermore, it was not detected in 100 unrelated control individuals, so the involvement of PORCN in our patient with the clinical suspicion of GGS was supported by the identification of the missense mutation within the transcribed PORCN sequences exchanging a conserved amino acid.

After the identification of the PORCN mutation in the patient, her parents also underwent genetic screening for the mutation research, which yielded negative results. During her clinical history, she also showed dental anomalies, hair shaft anomalies, orbital and endocranial calcifications, alternate light–dark patches over the extremities and trunk, atrial septal defect, mild pulmonary hypertension, urinary and abdominal disturbances, mild developmental delay, and short stature. Surgical history included several orthopedic interventions for skeletal abnormalities. During a cardiological checkup, a thyroid nodule was found in the right lobe, for which she was referred to our center.

## Diagnostic assessment, therapeutic intervention, follow-up, and outcome

**Figure 1:**
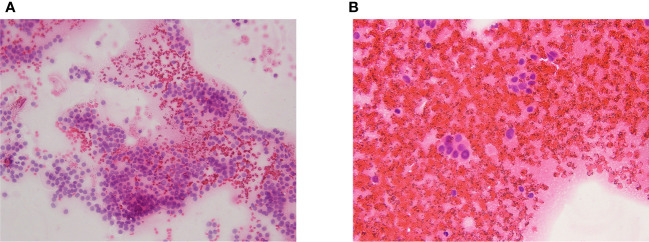
**(A, B)** The cytological exam showed cells with nuclear atypia, nuclear inclusions and rare grooves arranged in micro follicles, and foam histiocytes.

A diagnostic workup was started. TSH levels were normal (1.4 mcIU/mL), calcitonin, TPO-Abs, and Tg-Abs levels were undetectable, but neck ultrasound showed a 17-mm solid, hypoechoic nodule in the middle of the right lobe, in the context of a multinodular goiter [right lobe: 1.3 (transverse) × 1.4 (anteroposterior) × 4.2 (longitudinal) cm, volume = 4 mL; left lobe: 1.2 (transverse) × 1.2 (anteroposterior) × 3.9 (longitudinal) cm, volume = 2.9 mL]. The ultrasound did not show neck lymph nodes suspicious for malignancy. A thyroid fine needle aspiration biopsy was therefore performed; at the cytological examination, the smear showed cells with nuclear atypia, nuclear inclusions and rare grooves arranged in micro follicles, and foam histiocytes. The cytology sample was suspicious of malignancy (Thy4, according to the British Thyroid Association classification system 2014). **Figures 1A, B** depict the main cytological features. Molecular analyses on the cytology sample were negative for gene mutations (N-RAS, K-RAS, H-RAS, and bRAF). The genetic analysis was performed either on cytological specimens or on histological specimens after surgery.

**Figure 2:**
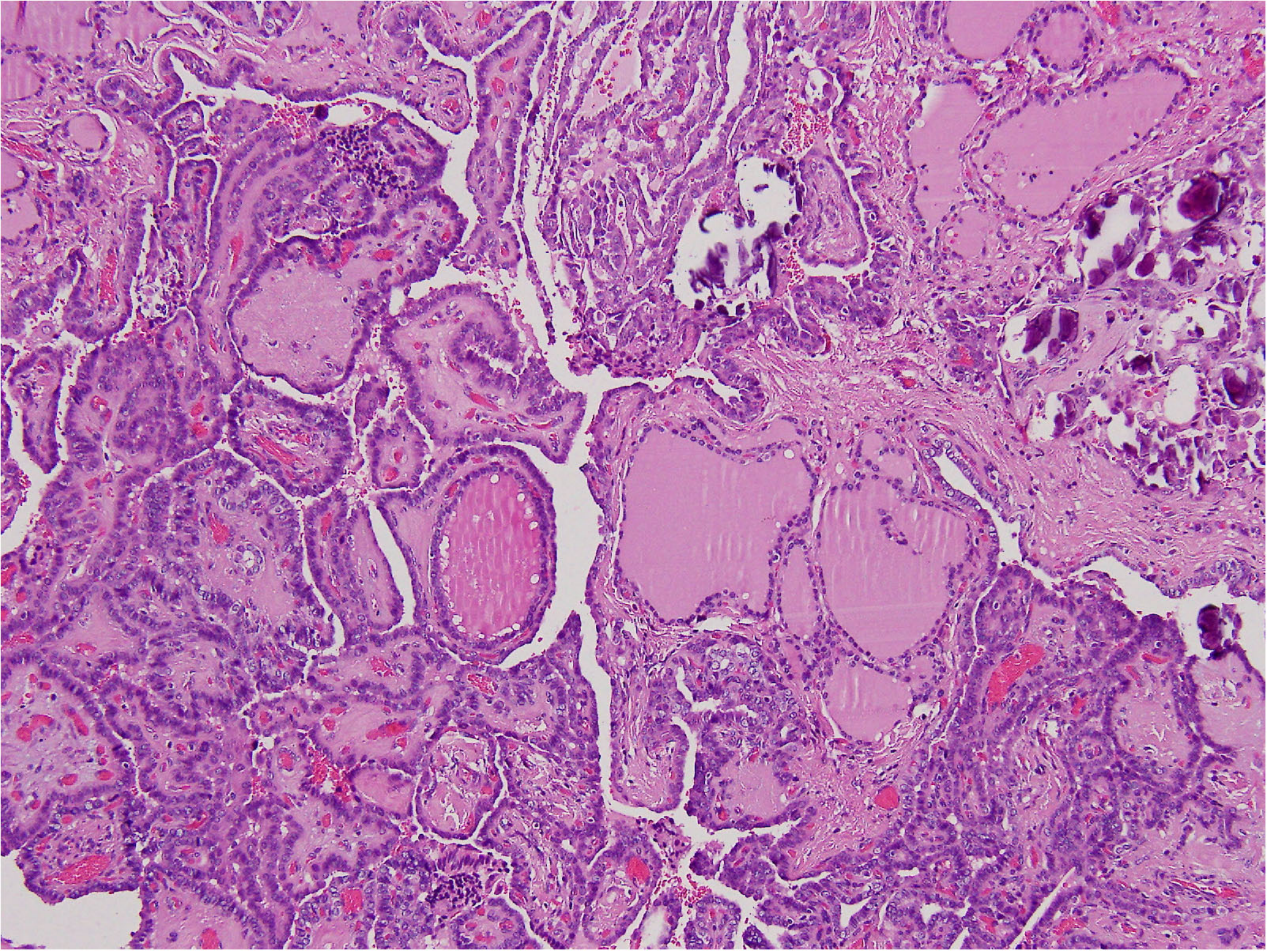
The histological exam disclosed multifocal, bilateral, classic PTC.

In August 2022, the patient underwent total thyroidectomy plus lymphadenectomy of the right central compartment (level VI). The operation was complicated by a neck hematoma that was treated by topical therapy. The scar healed very slowly and a broad-spectrum antibiotic therapy was given. The histological exam disclosed multifocal, bilateral, classic PTC (**Figure 2**), infiltrating the tumor capsule, and micrometastases (i.e., metastases less than 0.2 mm) in five lymph nodes (5+PTC/7). The tumor infiltrated the thyroid right lobe, but neither thyroid capsule nor vessel invasion was detected. According to the TNM AJCC classification system, 8th edition, the neoplasm was classified as pT1bm, pN1a, cM0. Following thyroidectomy, serum thyroglobulin concentrations were elevated at 2.3 ng/mL. Thus, the patient was submitted to radioiodine (131I) ablation therapy after administration of recombinant human thyrotropin (Thyrogen, 0.9 mg I.M., once a day for two consecutive days). Thyroglobulin autoantibodies were undetectable either before or after surgery. After 3 and 6 months, 131I, serum thyroglobulin concentrations were undetectable, and neck US did not demonstrate residual thyroid tissue or suspicious neck lymph nodes. The patient is regularly followed up, without disease recurrence (**Table 2**).

**Table 2:** Sections summary and timeline of the reported case.

Introduction	Goltz–Gorlin syndrome (GGS) is a rare X-linked disorder caused by PORCN gene mutations and characterized by several abnormalities. It encompasses a heterogeneous presentation depending on gene expression and mosaicism.
Case description	A 16-year-old female patient, born with multiple congenital dysmorphisms consistent with GGS and confirmed by genetic exam (2007), was diagnosed with a thyroid nodule (June 2022).
Diagnostic assessment	A neck ultrasound showed a 17-mm solid, hypoechoic nodule in the right lobe. A thyroid fine needle aspiration biopsy was performed, and the cytology sample was suspicious of malignancy (Thy4) (July 2022).
Treatment and follow-up	The patient underwent total thyroidectomy plus lymphadenectomy of the right central compartment (level VI) (August 2022). The histological exam disclosed a papillary thyroid carcinoma (PTC) with lymph node micrometastases (pT1bm, pN1a, cM0). Radioiodine therapy was performed. At 3- and 6-month follow-up, the patient did not present either ultrasound or laboratory PTC recurrence (November and February 2022).
Discussion	The PORCN gene is involved in the secretion and signaling of Wnt proteins, which contribute to tissue homeostasis by controlling proliferation, stem cell activation, and self-renewal. Wnt proteins are implicated in tumor growth in Wnt-dependent tissues, including the thyroid gland.
Conclusions	The early onset of PTC in a GGS patient, in the absence of other variants strongly associated with familial cancers, hints at the possibility that PORCN mutation may be a contributing factor in the susceptibility to thyroid cancer in this case.

## Discussion

GGS is a very rare condition. Approximately one person in a million is affected, but the exact prevalence is unknown (3). This syndrome is named after Goltz, who reported three female patients with FDH in 1962. Afterward, in 1963, Gorlin et al. gathered an extensive review of 11 previously published reports, going up to the first described by Jessner in 1921. The predilection for female patients is strong (the female–male ratio 9:1), while no racial or ethnic predilection has been documented. Approximately 95% of female patients with GGS have a *de novo* pathogenic variant in the PORCN gene and only 5% inherit it from a parent (3). An affected heterozygous woman has a 50% potential to transmit the PORCN pathogenic variant, but the effective sex ratio of offspring is 33% unaffected female offspring, 33% affected female offspring, and 33% unaffected male offspring, considering that the majority of male conceptuses with a PORCN pathogenic variant are presumed to be spontaneously aborted. If the affected female patient is mosaic for a PORCN pathogenic variant, the risk to her female offspring of inheriting the pathogenic variant is related to the level of mosaicism in her germline, up to 50% (4). If the pathogenic variant in a family has been identified, prenatal testing for pregnancies at increased risk and preimplantation genetic testing should be performed. Prenatal testing remains the best strategy to provide valuable information to families about genetic disorders and congenital disabilities, as individuals affected by GGS can develop significant postnatal functional impairment in multiple organs and tissues.

Although it is very rare, GGS is now a well-recognized genetic syndrome. The genetics and molecular mechanisms leading to its development have been investigated. GGS is caused by mutations in the PORCN gene (Xp11.23), which encodes porcupine O-acyltransferases, an endoplasmic reticulum transmembrane protein involved in the secretion and signaling of wingless proteins (Wnt) (5). Missense, synonymous, loss-of-function mutations of the PORCN gene have been documented (6). The phenotypes are highly variable due to tissue mosaicism; female patients usually have random X-chromosome inactivation (functional mosaicism), while male patients nearly always have postzygotic somatic mosaicism. Almost all affected male patients have somatic mosaicism for a PORCN pathogenic variant and are generally more mildly affected than female patients and so do not come to medical attention until adulthood (4). Furthermore, fathers with FDH are typically more mildly affected than their daughters, and this discrepancy would be attributed to mosaicism in male patients (4).

In our patient, a *de novo* heterozygous missense pathogenic variant (c.1250T>C; p.F417S) was detected. After consulting ClinVar, Varsome, Franklin Uniprot, Ensembl, OMIM, gene2phenotype, Gene Cards, gnomAD, PanelApp, CGD, ClinGen, and PubMed, we excluded other reported cases with this pathogenic variant in the current literature (5, 6).

Our patient displayed most of the GGS characteristics. She had congenital skin abnormalities, in particular, dermal hypoplasia and—in the circumscribed area—even cutis aplasia, besides telangiectasias. These skin alterations cause itching, pain, and irritation, and could lead to skin infections. Interestingly, our patient experienced two post-surgical complications, probably attributable to her underlying disease: a neck hematoma and a slow healing of the surgical wound despite broad-spectrum antibiotics.

As already mentioned, the PORCN gene is involved in the secretion and signaling of Wnt proteins. Wnt proteins are a family of highly conserved secreted cysteine-rich glycoproteins and contribute to tissue homeostasis by controlling proliferation, stem cell activation, and self-renewal (5). Since the discovery by Nusslein-Volhard and Wieschaus in 1980, the most extensively studied Wnt pathway is the Wnt/β-catenin pathway, due to its important role in cancer initiation and progression (7). This pathway physiologically plays a fundamental role in epithelial renewal and development.

The Wnt pathway acts through the activation of two alternative branches: the canonical and the non-canonical pathway (8). The activation of the non-canonical pathway, not dependent on β-catenin-driven transcription, relies on changes that affect the cytoskeletal organization and calcium homeostasis, and it is mainly related to cell differentiation, polarity, and migration (9). On the other hand, the canonical Wnt/β-catenin pathway has been widely studied as a classical signaling and highly conserved pathway. As it is a ligand-dependent activation, when the Wnt ligands are absent, the cytoplasmic β-catenin is bound and phosphorylated by the APC/Axin/CK1α/GSK3β destruction complex. It is followed by degradation through the ubiquitin–proteasome pathway, which suppresses the transcription of Wnt target genes, such as Axin2, C-Myc, CyclinD1, and Survivin. Instead, when the Wnt ligands are present, the Wnt proteins are bound to their receptors, named Frizzled and Lrp5/6 (10). This protein–receptor binding stimulates the phosphorylation of both LRP6 and Dvl and after that it disintegrates the destruction complex, resulting in the accumulation of cytoplasmic β-catenin and in the initiation of specific gene transcription after translocating to the nucleus (11–13). The palmitoylation of Wnt proteins mediated by PORCN is an essential modification for their correct secretion and binding to the Frizzled receptors (10). Biochemical studies confirmed that the addition of the palmitoleate to a highly conserved serine residue in Wnt is catalyzed by PORCN (14, 15). This palmitoylation is fundamental to the secretion of Wnts and the binding with receptors to activate Wnt signaling (16–18). Wnt palmitoylation, secretion, and signaling can be abolished by the substitution of this conserved serine residue with an alanine or a cysteine (19). Furthermore, PORCN is the only enzyme that catalyzes Wnt palmitoylation without influencing other lipid-modified signaling proteins (20, 21). It implies that PORCN inhibitors are highly specific to block the Wnt pathway, so targeting PORCN to halt the aberrant Wnt signaling is a rising and attractive therapeutic strategy in cancers (10).

The aberrant regulation of the Wnt pathway has been associated with bone anomalies, neurodevelopmental disorders, and cardiovascular diseases, among other diseases, and also the occurrence and development of cancer. The Wnt/β-catenin pathway is involved in tumor growth in Wnt-dependent tissues such as skin or colon, but it is accepted that inappropriate Wnt signaling also sets in the thyroid. Some components of this pathway, such as β-catenin and Axin, are often mutated in thyroid cancer (22). The alterations in Wnt signaling occur as a late event in thyroid cell transformation that affects anaplastic thyroid tumors; recent data suggest that it also happens in PTC (22). Ivanova et al. (23) found higher expression of β-catenin in PTC than in other tumors, while Dai et al. (24) underlined the link between inappropriate Wnt signaling and PTC, to the point that they proposed, as a new possible therapeutic target, an exosomal long non-coding RNA acting on the Wnt/β-catenin pathway, which determined an aggravation in PTC progression and invasion. Despite previous studies, there is still a lack of data regarding any known regular or conditional expression in specific components of thyroid tissue.

In GGS, the tendency to develop tumors arranged in papillae has been amply documented in the literature. Papillomas of the oral mucosa, nose, pharynx, esophagus, larynx, trachea, stomach, eyelid, groin, and distal extremities are reported and already included among clinical manifestations. As well as in other GGS-related tumors, we can speculate that Wnt/β-catenin pathway alterations induced by PORCN mutation could lead to the formation of PTC.

This case is unique as we described PTC in a patient affected by GGS with a new pathogenetic variant. In our patient, a spinal defect previously undescribed in patients with GGS was reported in the first year of life (6). Subsequently, during childhood, the patient developed other clinical findings classically associated with GGS. It could probably signify that PORCN mutation clinical manifestations are gradually revealed and, presumably, that the case is still evolving.

The fact that our patient has developed a PTC at 16 years of age, in the absence of variants strongly associated with familial cancers, hints at the possibility that PORCN mutation may contribute to the susceptibility to thyroid cancer. PTC may or may not be associated with the PORCN pathogenic variant but, in any case, considering that it is a new variant, we recommend caution for patient carriers of the same pathogenic variant in the future, suggesting ultrasound thyroid screening for early detection of cancer.

Even if thyroid cancer is rare in the pediatric population, it remains the leading cause of pediatric endocrine cancer. Pediatric thyroid carcinomas carry a unique set of molecular, pathologic, and clinical characteristics. Children more often than adults present with advanced-stage disease. The cause could be sought in biological and molecular differences between pediatric and adult thyroid cancer. In particular, PTC, which accounts for approximately 90% of pediatric thyroid cancer, often presents gene fusions that influence the histologic subtypes (25). Pediatric thyroid tumors are typically associated with more aggressive disease and extensive extrathyroidal spread, but, fortunately, these molecular peculiarities offer unique options for targeted medical therapies. Differences are also identified in pediatric follicular thyroid cancer, despite the paucity of studies published in the literature on pediatric non-papillary thyroid tumors, due to their rarity, and on medullary carcinoma, most frequently diagnosed in the pediatric population in the setting of prophylactic thyroidectomies for known multiple endocrine neoplasia syndromes (MEN-2). The knowledge of histotypes and underlying molecular alterations common in pediatric thyroid cancer is important as it may directly affect diagnostic test selection, therapeutic recommendations, and long-term outcomes (25).

## Conclusions

GGS is an X-linked dominant and congenital dysmorphic disorder characterized by several abnormalities. It is caused by mutations in the PORCN gene, involved in the secretion and signaling of Wnt proteins, and implicated in tumor growth in Wnt-dependent tissues, including the thyroid gland. Our case reports for the first time a PTC in a patient affected by GGS.

The early onset of PTC in our patient with GGS, in the absence of variants strongly associated with familial cancers, hints at the possibility that PORCN mutation may be a contributing factor in the susceptibility to thyroid cancer in this case. It may also suggest the precaution of submitting these patients to a careful follow-up, in order to improve long-term outcomes in these young patients. Further studies are needed to corroborate the relationship between GGS and PTC.

## Data availability statement

The raw data supporting the conclusions of this article will be made available by the authors, without undue reservation.

## Ethics statement

Written informed consent was obtained from a legally authorized representative(s) for anonymized patient information to be published in this article.

## Author contributions

GP: responsible for the endocrine management of the patient. SC: responsible for the histological examination. FC: wrote the original draft of the manuscript. GP and AP: wrote and edited the final manuscript. All authors reviewed and approved the final draft.
